# Regulatory relationships among *aldB*, *ampH*, and *acoR* and their impact on β-lactam susceptibility in *Phytobacter diazotrophicus*

**DOI:** 10.3389/fmicb.2025.1687374

**Published:** 2025-11-11

**Authors:** Jiansheng Lin, Jingyang Zheng, Gaoxiong Wang

**Affiliations:** 1 Microbiology Laboratory, Quanzhou Women's and Children's Hospital, Quanzhou, China; 2 The Affiliated Women’s and Children's Hospital of Huaqiao University, Quanzhou, China; 3 Respiratory Department, Quanzhou Women’s and Children’s Hospital, Quanzhou, China; 4 Research Administration Office, Quanzhou Women's and Children's Hospital, Quanzhou, China

**Keywords:** *Phytobacter diazotrophicus*, *aldB*, *ampH*, *acoR*, antibiotic resistance

## Abstract

**Background:**

*Phytobacter diazotrophicus* (*P. diazotrophicus*) is an emerging opportunistic pathogen responsible for various human infections. However, it remains unclear whether aldehyde dehydrogenase B (*aldB*) mediates antibiotic resistance in *P. diazotrophicus*, and whether acetoin catabolism operon regulator (*acoR*) gene is associated with antibiotics. This study aims to explore the association between the *aldB* gene and bacterial susceptibility to β-lactam antibiotics, investigate the potential mechanism by which *aldB* mediates antibiotic resistance, and clarify the regulatory mechanism of *aldB* by the upstream adjacent gene *acoR*.

**Methods:**

Gene knockout was performed using homologous recombination. The minimum inhibitory concentrations (MICs) of 12 β-lactam antibiotics were determined using the MH agar plate dilution method. RNA transcriptome analysis was performed on the wild-type and *aldB* knockout strains of *P. diazotrophicus* Pd1. mRNA expression levels were measured using real-time quantitative polymerase chain reaction (qPCR). The binding of the purified AcoR-HTH protein to P*aldB* was analyzed using electrophoretic mobility shift assays (EMSA). Ceftazidime was used for antibiotic stimulation tests.

**Results:**

Compared with the wild-type strain, the *aldB* knockout strain exhibited significantly increased MICs for carbenicillin, cefalotin, cefoxitin, cefuroxime, cefotaxime, ceftazidime, cefepime, and aztreonam by at least 2-, 2-, 2-, 4-, 4-, 4-, 2-, and 4-fold, respectively. RNA transcriptome sequencing revealed that *ampH* was most significantly downregulated in the *aldB* knockout strain, which was confirmed by qPCR. Compared with the wild-type strain, the *ampH*-knockout strain exhibited significantly increased MICs for carbenicillin, piperacillin, cefalotin, cefoxitin, cefuroxime, cefotaxime, ceftazidime, cefepime, aztreonam, and ertapenem by at least 2-, 4-, 8-, 4-, 4-, 4-, 4-, 4-, 4-, and 2-fold, respectively. Compared with the wild-type strain, the *acoR* knockout strain exhibited significantly increased MICs for carbenicillin, piperacillin, cefalotin, cefoxitin, cefuroxime, cefotaxime, ceftazidime, cefepime, aztreonam, and ertapenem by at least 2-, 4-, 4-, 8-, 4-, 8-, 8-, 4-, 4-, and 2-fold, respectively. Compared with the wild-type strain, the *acoR* knockout strain significantly downregulates the mRNA expression of *aldB*. The sequence ACGACACAGTTCGCGAA was identified as a recognition site for AcoR in *P. diazotrophicus* through software alignment and EMSA experiments. Compared with the untreated wild-type strain, *aldB* mRNA expression levels in the ceftazidime-stimulated wild-type strain reduced significantly.

**Conclusion:**

In *P. diazotrophicus*, *aldB* and *acoR* reduced some β-lactam resistance by facilitating *ampH* and *aldB* expression, respectively. This is the first report that links *acoR* to β-lactam antibiotics and demonstrates that AcoR positively regulates *aldB*. Under ceftazidime stress, *P. diazotrophicus* reduced *aldB* expression to increase its tolerance to the antibiotic. The discovery of the mechanism by which AcoR regulates *aldB* expression provides preliminary evidence for subsequent research on drug resistance mechanisms.

## Introduction

*Phytobacter diazotrophicus* (*P. diazotrophicus*), belonging to the family Enterobacteriaceae, is an emerging opportunistic pathogen associated with various human infections (sepsis and biliary tract infections) (Lin et al., 2024; Smits et al., 2022) and occasionally causes fatal outbreaks (Pillonetto et al., 2018). The clinical significance of *P. diazotrophicus* has likely been underestimated due to frequent misidentification of clinical isolates as other Enterobacteriaceae. β-lactam antibiotics are commonly used to treat bacterium-induced infections. Over the past 2 years, clinical reports have confirmed the emergence of multidrug-resistant *P. diazotrophicus* strains (Hon et al., 2023; Almuzara et al., 2024). Therefore, assessing drug resistance mechanisms in *P. diazotrophicus* is crucial for the treatment and prevention of clinical infections.

The intergenic region between aldehyde dehydrogenase B (*aldB*) and *yiaW* may contribute to the tolerance of *Escherichia coli* to antimicrobial agents rather than the *aldB* itself (Hu and Coates, 2005). However, *aldB* gene reduces the resistance of *Lactobacillus reuteri* (*L. reuteri*) to chloramphenicol but not to β-lactam antibiotics (Wang et al., 2024). This study did not assess why *aldB* reduced chloramphenicol resistance. The presence of *aldB* increases the number of persisters under non-stress conditions. However, the effect of *aldB* on persister counts is environmentally dependent. For example, under amino acid limitation, *aldB* knockdown unexpectedly increased persister numbers. Because this increase is associated with glycolytic metabolites, metabolic flux may play a crucial role in *aldB*-mediated persister formation (Kawai et al., 2018). Therefore, it can be concluded that the *aldB* regulates antibiotic resistance in certain bacteria (but not all) and is involved in persister formation.

In *E. coli*, low-molecular-mass penicillin-binding proteins (LMM PBPs) are crucial for the proper formation of cell morphology. These enzymes possess DD-carboxypeptidase and/or DD-endopeptidase activities that are associated with the maturation and remodeling of peptidoglycan (PG) (Sauvage et al., 2008). AmpH is an AmpH-type class C LMM PBP (Sauvage et al., 2008) involved in PG recycling as a bifunctional enzyme with both DD-endopeptidase and DD-carboxypeptidase activities (González-Leiza et al., 2011). Although AmpH is closely associated with ampicillin class C β-lactamase (AmpC) and other class C β-lactamases, it did not exhibit β-lactamase activity toward nitrocefin in previous studies (Henderson et al., 1997). However, AmpH can strongly bind to penicillin G, cefoxitin, and cephalosporin C (Henderson et al., 1997). Additionally, AmpH exhibits weak β-lactamase activity against nitrocefin and is a penicillin-binding protein owing to its affinity for β-lactam substances (such as the fluorescent penicillin Bocillin FL and cefmetazole) (González-Leiza et al., 2011). It is demonstrated that AmpH consistently binds to certain β-lactam antibiotics. Genetically, *ampH* has not yet been knocked out to assess its association with β-lactam antibiotics.

In the genome of *P. diazotrophicus* Pd1, acetoin catabolism regulator (*acoR*) gene is located directly upstream of *aldB* and is divergently transcribed relative to it. *acoR* encodes a sigma-54-dependent transcriptional regulatory protein of factor for inversion stimulation (Fis) family. Fis is a versatile DNA-binding protein that plays a crucial role in coordinating global bacterial gene expression in response to growth phase and environmental stress. It inhibits *aldB* expression (Xu and Johnson, 1995) and enhances bacterial resistance to ciprofloxacin by downregulating genes involved in pyocin biosynthesis (FLong et al., 2020). Additionally, Fis acts as an accessory transcriptional activator at the promoter of multiple antibiotic-resistant *marRAB* operon (Martin and Rosner, 1997). The acetoin catabolic pathway, which is significant in numerous microorganisms, is transcriptionally regulated by *acoR* (Peng et al., 2020). It remains unclear whether *aldB* mediates antibiotic resistance in *P. diazotrophicus*, and whether *acoR* is associated with antibiotics.

In this study, we report that *aldB* mediates antibiotic resistance in *P. diazotrophicus* by affecting *ampH* expression. Additionally, we demonstrated the association between *acoR* and antibiotics, its regulatory role in *aldB*-mediated antibiotic resistance, and the role of *aldB* under ceftazidime stimulation. This study aims to elucidate the mechanism by which AcoR regulates *aldB* in *P. diazotrophicus*, thereby providing a theoretical basis for the prevention and control of related infections.

## Materials and methods

### Bacterial strains and culture conditions

*Phytobacter diazotrophicus* Pd1, which was isolated from the blood of a newborn with D-galactosemia complicated by sepsis, was preserved in our laboratory (Lin et al., 2024). The recombinant pKAS32 plasmid carrying the tetracycline resistance gene cassette was purchased from Baosai Biotechnology Co., Ltd., Hangzhou, China. The pDM4 plasmid was purchased from Wuhan Miaoling Biotechnology Co., Ltd. and used for constructing suicide plasmid. *E. coli* S17-1 (λpir) was purchased from Beyotime Biotechnology and provides a host environment for the pDM4 plasmid. *P. diazotrophicus* Pd1 was cultured on a blood agar plate at 37 °C for 24 h.

### Construction of deletion mutant strains

Construction of *aldB* gene knockout based on homologous recombination method (Lin et al., 2025). The genome of *P. diazotrophicus* Pd1 was extracted in accordance with the requirements of TIANamp Bacteria DNA Kit from Tiangen Biochemical Technology Co., Ltd. The upstream and downstream arms of *aldB* were amplified from *P*. *diazotrophicus* Pd1 genomic DNA by high-fidelity PCR using the primer pairs *aldB*-up-F/*aldB*-up-R and *aldB*-down-F/*aldB*-down-R, respectively. The tetracycline resistance gene was amplified from the pKAS32 plasmid by high-fidelity PCR using the primer pair *aldB*-TC-F and *aldB*-TC-R. High-fidelity PCR was purchased from Vazyme. The total reaction volume of 50 μL consisted of 20 μL ddH2O, 25 μL 2 × Phanta Max Master Mix, 2 μL primer F, 2 μL primer R, and 1 μL template DNA. The thermal cycle was programmed for 3 min at 95 °C for pre-denaturation, followed by 32 cycles of 15 s at 95 °C for denaturation, 15 s at 60 °C for annealing, 60 s at 72 °C for extension; a final extension was conducted for 5 min at 72 °C (Lin et al., 2025). The above three fragments were ligated into the Suicide pDM4 plasmid using the ClonExpress MultiS One Step Cloning Kit (Vazyme Biotech Co., Ltd) with LIC (Ligation-Independent Cloning). The recombinant pDM4 plasmid was verified for ligation by Sanger sequencing using the primer pairs pDM4-sequence-F and pDM4-sequence-R. The recombinant pDM4 plasmid was electroporated into competent *P. diazotrophicus* Pd1 using an electroporation instrument (1.8 kV, 400 *Ω*). Subsequently, the bacterial suspension was spread on LB agar plates containing tetracycline (20 μg/mL) and piperacillin (24 μg/mL). Single colonies were picked and subcultured for 5 generations. The knockout of the *aldB* gene was verified by PCR using the primer pair *aldB*-Delete-F and *aldB*-Delete-R. The primer sequences are listed in Table 1.

**Table 1:** Primer names and sequences in this subject.

Primer name	Primer sequence (5′-3′)
*aldB*-up-F	tgtggaatcccgggagagctcTGAAGCGGAACAGAACAGCC
*aldB*-up-R	acgacaagcttCATAAGATGTTCCTCTATTTTTTTATGGG
*aldB*-TC-F	catcttatgAAGCTTGTCGTGTTAAAGACCTCC
*aldB*-TC-R	taAAGCTTTTGGCGGGTGTCG
*aldB*-down-F	gacacccgccaaaagcttTAATACCACGTCTATTGCGTCAACA
*aldB*-down-R	cactagtggggcccttctagaAGCCCAGACCAAGCTGGTCG
pDM4-seqence-F	GTGACAATCACGAAACGCGG
pDM4-seqence-R	GGTATTTATTCGGCGCAAAGTGC
*aldB*-Delete-F	GCCAGACTGGCGCTAAAGTC
*aldB*-Delete-R	ATCCCAGGTTTCCGTCAGC
*ampH*-up-F	tgtggaatcccgggagagctcAAGCCGCGGTTTTGGTGA
*ampH*-up-R	cacgacaagcttCATCCGTAAATGAAAGAAGTGGATA
*ampH*-TC-F	tacggatgAAGCTTGTCGTGTTAAAGACCTCC
*ampH*-TC-R	cccggccaaatcaAAGCTTTTGGCGGGTGTCG
*ampH*-down-F	aaagcttTGATTTGGCCGGGCAGTT
*ampH*-down-R	cactagtggggcccttctagaAGCCATAAGTCGAAAATATAACGATG
*ampH*-Delete -F	CGCTCATATTCACGAAACGC
*ampH*-Delete -R	CTGGGTGTATATGGCACCGA
q-16S rRNA-F	CAGCCATGCCGCGTGTA
q-16S rRNA-R	TAATTCCGATTAACGCTTGCAC
q-*aldB*-F	CCTGTAGAAGGGCGCTGGT
q-*aldB*-R	GTTTTGCCCCACGCGTC
*acoR*-up-F	tgtggaatcccgggagagctcATGGGCGGCGTCGAGCGC
*acoR*-up-R	acgacaagcttCATGGCACACTCCGGAGAAC
*acoR*-TC-F	tgtgccatgAAGCTTGTCGTGTTAAAGACCTCC
*acoR*-TC-R	ttcaAAGCTTTTGGCGGGTGTCG
*acoR*-down-F	cacccgccaaaagcttTGAATTGCGCTTACAGGGGG
*acoR*-down-R	cactagtggggcccttctagaACGTACGACTACATCTCCTTCCTGA
*acoR*-Delete-F	CATTGCGGTTAAGGTAGTGTTCA
*acoR*-Delete-R	CTTCCCTGCCAACTGGAAGA
q-*ampH*-F	CGCTCATATTCACGAAACGC
q-*ampH*-R	CTGGGTGTATATGGCACCGA
acoR-HTH-F	CGGGATCCGCAGGTGGCCTCAGGGGCA
acoR-HTH-R	CCGCTCGAGGACGCTGTCGACGCTCTGG
P*aldB*-1-F	GCACACTCCGGAGAACTGAGAT
P*aldB*-1-R	CAAAACCTGTGCCGAACAGAG
P*aldB*-2-F	ATCCTGCCAGACAGCAATGAT
P*aldB*-2-R	TATGGGTACAGGTGAGGCGTC

Lowercase letters represent overlapping sequences. Underlines indicate restriction enzyme cleavage sites.

The construction process of the *ampH* knockout strain is consistent with that of the *aldB* knockout strain. High-fidelity PCR technology was used to amplify the upstream and downstream homologous arms and the tetracycline resistance gene cassette using the primer pairs *ampH*-up-F/*ampH*-up-R, *ampH*-down-F/*ampH*-down-R, and *ampH*-TC-F/*ampH*-TC-R, respectively. PCR verification was performed with the primer pair *ampH*-Delete-F/*ampH*-Delete-R to confirm the successful knockout of the *ampH* gene. All primer sequences are detailed in Table 1.

The construction of the *acoR* gene knockout strain also followed the method used for the *aldB* knockout strain. Amplification of the upstream homologous arm, downstream homologous arm, and tetracycline resistance gene cassette was accomplished using high-fidelity PCR with the primer pairs *acoR*-up-F/*acoR*-up-R, *acoR*-down-F/*acoR*-down-R, and *acoR*-TC-F/*acoR*-TC-R, respectively. The knockout efficiency of the *acoR* gene was confirmed by PCR verification using the primer pair *acoR*-Delete-F/*acoR*-Delete-R. The sequences of all related primers are detailed in Table 1.

### Antibiotic susceptibility test

The minimum inhibitory concentration (MIC) values of the strains against 12 β-lactam antibiotics, including carbenicillin, piperacillin, cefalotin, cefoxitin, cefuroxime, cefotaxime, ceftazidime, cefepime, aztreonam, ertapenem, meropenem, and imipenem, were determined using the MH agar plate dilution method. The specific procedure was as follows: first, the strains were revived on blood plates for 24 h, and single colonies were picked and subcultured for 24 h. Subsequently, each antibiotic with a serial dilution concentration was added to empty plates, and then 25 mL of MH agar medium at approximately 50 °C was poured into each plate. Before conducting the experiment, mark the regions for the wild-type strain and the knockout mutant strain on the bottom of each MH agar plate. Next, the concentration of the bacterial suspension was adjusted to the 0.5 McFarland turbidity standard using a McFarland turbidimeter with sterile 0.65% physiological saline, and 1 μL of the bacterial suspension was titrated onto the surface of the MH agar plates. After air-drying, the plates were placed in an incubator at 37 °C, and the MIC values of each strain were observed after 20 h. *E. coli* ATCC8739 was selected as the quality control bacterium. The MIC determination results of this reference strain fell within the range specified in the Clinical and Laboratory Standards Institute (CLSI) document M100-Ed35. This biological experiment was repeated three times.

### RNA transcriptome analysis

RNA transcriptome analysis was conducted on the *aldB* knockout strain and wild-type strain of *P. diazotrophicus* Pd1. Total RNA was extracted from the strains using the RNeasy Mini Kit (Qiagen, Hilden, Germany) following the manufacturer’s instructions, and the concentration and purity of the extracted RNA were determined using a Nanodrop 2000 spectrophotometer. RNA-seq paired-end sequencing was performed on the Illumina NovaSeq 6000 platform. Bioinformatics analysis of the sequencing data generated by the Illumina platform was carried out using the cloud computing platform (cloud.majorbio.com) provided by Shanghai Majorbio Bio-pharm Technology Co., Ltd. After obtaining the read counts for each gene, differential expression analysis was performed using DESeq2 software with reference to the *P. diazotrophicus* Pd1 genome. The criteria for screening differentially expressed genes were as follows: an adjusted *p*-value < 0.05, and a fold change (FC) in gene expression between samples of ≥2 (up-regulated) or ≤0.5 (down-regulated).

### RNA isolation and quantitative PCR

Total RNA was extracted using FreeZol Reagent (Vazyme Biotech Co., Ltd., Nanjing, China), with operations strictly following the manufacturer’s instructions. The specific steps were as follows: 1 mL of bacterial suspension was placed in an EP tube and centrifuged at 11,200 rpm for 3 min. After discarding the supernatant, 100 μL of lysozyme reagent (30 mg/mL) was added to resuspend the bacterial pellet, followed by pipetting to mix thoroughly, and incubation at 37 °C for 30 min. After this treatment, 500 μL of FreeZol Reagent was added, and the mixture was vortexed thoroughly to mix well, then left to stand at room temperature for 5 min. Subsequently, it was centrifuged at 11,200 rpm for 15 min at room temperature, and the upper aqueous phase was carefully aspirated. An equal volume of isopropanol was added, and the mixture was gently inverted to mix well, then allowed to stand at room temperature for 10 min. It was centrifuged again at 11,200 rpm for 10 min at room temperature, and the supernatant was discarded. One milliliter of 75% ethanol was added, the EP tube was inverted 5 times, and centrifugation was performed at 9,100 rpm for 3 min at room temperature. The supernatant was discarded, and the steps of ethanol washing, centrifugation, and supernatant discarding were repeated once. The tube was left at room temperature for 3 min to allow the precipitate to air-dry naturally, and finally, 20 μL of RNase-free double-distilled water was added to dissolve the precipitate. After extraction, the RNA concentration was measured using a Nano-300 micro-spectrophotometer.

Five hundred nanogram of total RNA was taken, and cDNA was synthesized via reverse transcription using the HiScript III RT SuperMix for qPCR (+gDNA wiper) kit (Vazyme Biotech Co., Ltd., Nanjing, China). The specific steps were as follows: First, to remove genomic DNA, a 16 μL reaction system was prepared, containing 0.5 μL of RNA, 4 μL of 4 × gDNA Wiper Mix, and 11.5 μL of RNase-free double-distilled water. After mixing, it was incubated at 42 °C for 2 min. Subsequently, 5 μL of HiScript III RT SuperMix was added, and reverse transcription was performed under the following conditions: incubation at 37 °C for 15 min, followed by treatment at 85 °C for 5 s.

The real-time fluorescent quantitative PCR (qPCR) reaction was performed using 2.5 ng of cDNA as the template, with ChamQ Blue Universal SYBR PCR Master Mix (Vazyme Biotech Co., Ltd., Nanjing, China) on the Gentier 96 fully automated real-time fluorescent quantitative PCR analysis system (Tianlong Technology Co., Ltd., Xi’an, China). The 20 μL reaction system consisted of 10 μL of 2 × ChamQ Blue Universal SYBR qPCR Master Mix, 0.4 μL of forward primer (10 μM), 0.4 μL of reverse primer (10 μM), 1 μL of cDNA, and 8.2 μL of double-distilled water. The qPCR reaction conditions were: pre-denaturation at 95 °C for 30 s; 40 cycles of denaturation at 95 °C for 10 s and annealing-extension at 60 °C for 10 s; finally, melting curve analysis was performed according to the instrument’s default program. Detailed information on the primers used is provided in Table 1. To validate qPCR primer efficiency, the efficiency (E) is calculated using the formula *E* = [10^(−1/slope) - 1] × 100%. A primer is considered to have qualified efficiency if 90% ≤ *E* ≤ 110%; if the *E* value falls outside this range, the primer needs to be optimized. Data normalization was performed using the housekeeping gene 16S rRNA as an internal reference, and the relative expression levels of the *ampH* and *aldB* genes were calculated using the 2^(−ΔΔCt) method. For the qPCR experiment, five biological replicates were selected for each group, and the Cohen’s d value was set to > 0.8.

### Analysis of AcoR binding sites

In this study, the sequence upstream of the translation initiation site of the *aldB* gene in *P. diazotrophicus* was aligned with the reported promoter sequence bound by the AcoR protein using DNAMAN software to analyze the potential binding site of AcoR.

### Expression and purification of the helix-turn-helix domain of AcoR

The DNA fragment of the acoR gene, which encompasses its helix-turn-helix (HTH) domain, was amplified via PCR from the genomic DNA of *P. diazotrophicus* Pd1. The PCR primers used for this amplification were acoR-HTH-F and acoR-HTH-R. Following amplification, the PCR product was subjected to restriction enzyme digestion and subsequently ligated into the expression vector pET21b. The resulting recombinant plasmid, designated as pET-HTH, was transformed into *E. coli* strain BL21 (DE3) to generate the recombinant strain BL21 (pET-HTH). Finally, the BL21 (pET-HTH) strain was cultured in LB medium until it reached the exponential growth phase. Expression and purification of the HTH-His protein was performed as previously described (Lin et al., 2025). Before purification, the inclusion body-borne HTH-His protein was solubilized in a 50 mM Tris–HCl buffer (pH 8.0) containing 8 M urea, following 20 min of ultrasonic disruption. The lysate was centrifuged at 12,000 rpm for 20 min, and the supernatant was collected. After purification, dialysis bags filled with the protein-containing supernatant were placed in the following dialysates, respectively, for dialysis: 50 mM Tris–HCl buffer (pH 8.0) containing 100 mM NaCl, 1 mM DTT, 0.02% Tween 80 and with urea concentrations of 6 M, 4 M, 2 M respectively, as well as PBS buffer (pH 7.35). Dialysis was performed at 4 °C, and the dialysate needed to be replaced after 6 h of dialysis each time.

### Electrophoresis mobility shift assays

Promoter region P*aldB*-1 was designed to contain the potential AcoR-binding site (sequence: ACGACACAGTTCGCGAA). To further verify whether this sequence functions as a binding site, promoter region P*aldB*-2 was constructed to lack this specific motif. Promoter regions P*aldB*-1 and P*aldB*-2 from *P. diazotrophicus* Pd1 genome were PCR amplified using primer pairs P*aldB*-1-F/P*aldB*-1-R and P*aldB*-2-F/P*aldB*-2-R, respectively. The concentrations of P*aldB*-1 and P*aldB*-2 were determined using a Nano-300 micro-spectrophotometer. Electrophoretic mobility shift assays (EMSA) were performed as previously described (Peng et al., 2014), with minor modifications to analyze binding of the purified AcoR-HTH protein to P*aldB*. Finally, the non-denaturing electrophoresis gel was stained with SYBR™ Green for 1 h and imaged using a CLINX GenoSens 1,660 instrument.

### Ceftazidime stimulation test

Twenty-five microliter of bacterial suspension (either the wild-type strain or the *acoR* knockout strain) at a 0.5 McFarland standard was inoculated into 25 mL of Brain Heart Infusion (BHI) medium and incubated for 24 h at 37 °C. For the dose-dependent experiment, ceftazidime should be individually added to each bacterial suspension in the experimental groups to adjust the final concentration to 0.125, 0.25, 0.5, and 1 μg/mL, respectively, (corresponding to 1×, 2×, 4×, and 8 × MIC). After mixing well, the suspensions are incubated statically at 37 °C for 1 h. For the time kinetic experiment, ceftazidime should be individually added to each bacterial suspension in the experimental groups to adjust the final concentration to 0.25 μg/mL (equivalent to 2 × MIC). After mixing well, the suspensions are incubated statically at 37 °C for 0.5, 1, 2, and 3 h, respectively. Meanwhile, under the same conditions, the untreated wild-type strain and untreated *acoR* knockout strain (without ceftazidime exposure) served as control groups. Since ceftazidime was dissolved in sterile double-distilled water, and the volume of ceftazidime added to the experimental group was extremely small, which is negligible relative to the 25 mL culture medium, it is unnecessary to set up a solvent control group.

Next, 1 mL aliquots of each bacterial suspension (both experimental and control groups) were centrifuged at 6,000 rpm for 3 min. The supernatant was carefully aspirated, and the bacterial pellet was washed three times with PBS buffer (pH 7.35) to remove residual medium and antibiotics. Finally, bacterial RNA isolation and qPCR were performed following the procedures described above. This biological experiment was independently replicated five times to validate the reproducibility of the results.

### Statistical analysis

In this study, the data of all sample groups were verified to exhibit a normal distribution via the Shapiro–Wilk test, and the homogeneity of variances between groups was confirmed through the Levene test. Based on the above prerequisite conditions, Student’s *t*-test was used to evaluate the statistical significance of differences between groups. A *p*-value < 0.05 was considered to indicate a statistically significant difference.

## Results

### *aldB* is associated with antibiotic susceptibility

To demonstrate the association between *aldB* and β-lactam antibiotics, we constructed an *aldB* deletion strain (Supplementary Figure S1) and determined the minimum inhibitory concentrations (MICs) of 12 β-lactam antibiotics in the wild-type and *aldB* knockout strains. Compared with wild-type *P. diazotrophicus*, the *aldB* knockout strain exhibited increased MICs for carbenicillin, cefalotin, cefoxitin, cefuroxime, cefotaxime, ceftazidime, cefepime, and aztreonam by at least 2-, 2-, 2-, 4-, 4-, 4-, 2-, and 4-fold, respectively (Table 2). These results indicate that *aldB* is associated with some β-lactam susceptibility and reduces *P. diazotrophicus* resistance to these antibiotics.

**Table 2:** The MIC values of antibiotics for wild-type strains and mutant strains.

Antibiotic	MIC (μg/mL)			
	WT	△*aldB*	△*ampH*	△*acoR*
Carbenicillin	256	>256	>256	>256
Piperacillin	32	32 (0)	128 (4)	256 (8)
Cefalothin	4	8 (2)	32 (8)	16 (4)
Cefoxitin	8	16 (2)	32 (4)	64 (8)
Cefuroxime	4	16 (4)	16 (4)	16 (4)
Cefotaxime	0.0156	0.0625 (4)	0.0625 (4)	0.125 (8)
Ceftazidime	0.125	0.5 (4)	0.5 (4)	1 (8)
Cefepime	0.0313	0.0625 (2)	0.125 (4)	0.125 (4)
Aztreonam	0.0313	0.125 (4)	0.125 (4)	0.125 (4)
Ertapenem	0.0156	0.0156 (0)	0.0313 (2)	0.0313 (2)
Meropenem	0.125	0.125 (0)	0.125 (0)	0.125 (0)
Imipenem	0.25	0.25 (0)	0.25 (0)	0.25 (0)

△aldB, △ampH, and △acoR indicate the aldB, ampH, and acoR knockout strains, respectively. The numbers in parentheses represent the fold increase.

### *aldB* reduces antibiotic resistance by upregulating *ampH*

**Figure 1:**
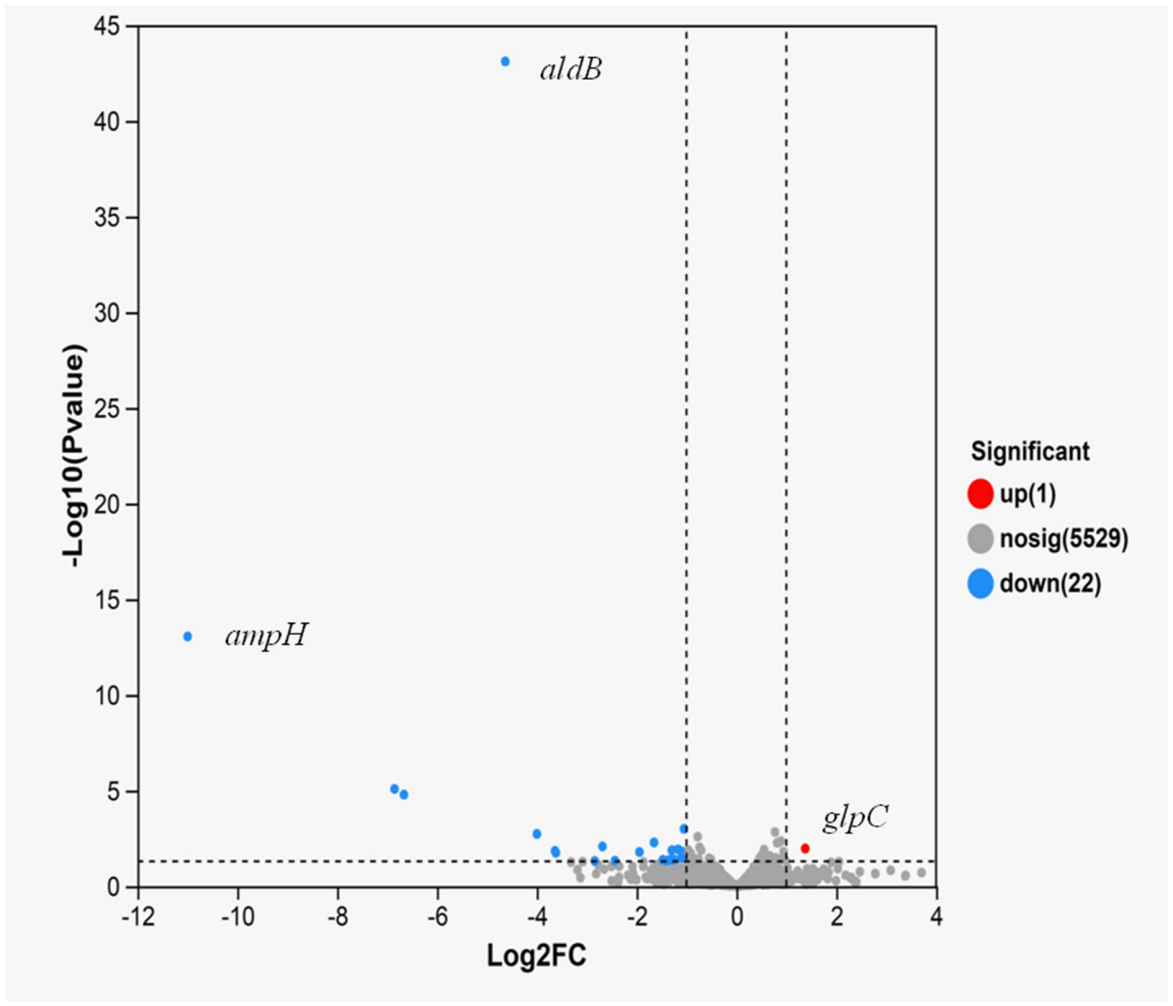
Volcano plot of expression difference for *aldB* knockout strain VS wild-type strain. The abscissa represents the fold change value of gene expression difference between the two groups of samples, i.e., the FC value. The ordinate represents the statistical test value of the difference in gene expression changes, i.e., the *p*-value. A higher *p*-value indicates a more significant expression difference, and both the abscissa and ordinate values have undergone logarithmic processing. Each point in the figure represents a specific gene: red points indicate significantly up-regulated genes, green points indicate significantly down-regulated genes, and gray points indicate genes with non-significant differences. After mapping all genes, it can be seen that the points on the left represent genes with down-regulated expression differences, and the points on the right represent genes with up-regulated expression differences. The points closer to the two sides and the top have more significant expression differences.

**Figure 2:**
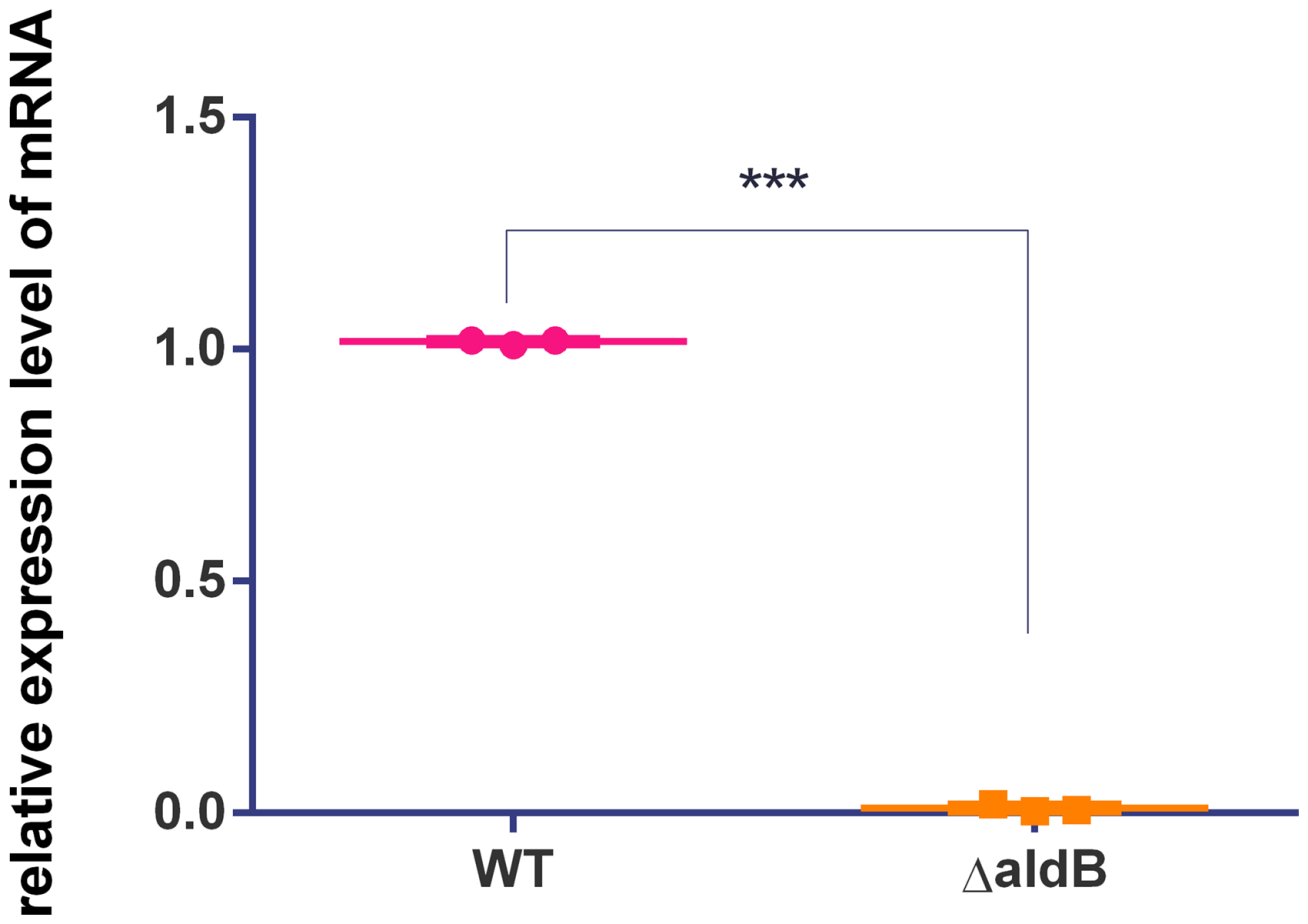
Comparison of relative expression levels of *ampH* between the wild-type strain and *aldB* knockout strain of *P. diazotrophicus*. Data represent the mean ± standard deviation of five sample results. *** indicates *p* < 0.001.

To assess how *aldB* reduces resistance to β-lactam antibiotics in *P. diazotrophicus*, transcriptome sequencing was conducted on both the wild-type and *aldB* knockout strains. Compared to wild-type *P. diazotrophicus*, the *aldB* knockout strain upregulated one gene and downregulated 22 genes (Table 3). Transcriptome analysis demonstrated that *aldB* was most significantly downregulated, confirming its successful knockout. Excluding *aldB*, *ampH* was most significantly down-regulated (Figure 1; Table 3). Real-time quantitative polymerase chain reaction (qPCR) was performed to confirm transcriptome sequencing results. The *E* values of 16S rRNA and *ampH* are 100 and 107% respectively, both falling within the ideal range (90–110%) for qPCR primer efficiency (Supplementary Figures S2, S3). Both the melting curves of 16S rRNA and *ampH* show a single peak, which indicates that the primers for 16S rRNA and *ampH* have good specificity (Supplementary Figure S4). *ampH* expression in the *aldB* knockout strain was reduced by approximately 100-fold than that in the wild-type strain (Supplementary Figure S5; Figure 2). This indicates that *aldB* upregulated *ampH* expression.

**Table 3:** Diferentially expressed genes identifed by RNA-seq.

Gene expression	Gene name	Gene function annotations	Fold change (△aldB/WT)
Down-regulated	*aldB*	Aldehyde dehydrogenase	0.04
	*ampH*	D-alanyl-D-alanine-carboxypeptidase/D-alanyl-D-alanine-endopeptidase	0
	–	Helix-turn-helix domain-containing protein	0.009
	–	Isochorismatase family protein	0.01
	–	Hypothetical protein	0.483
	*tcyB*	Amino acid ABC transporter permease	0.063
	*fliY*	Amino acid ABC transporter substrate-binding protein	0.318
	*metQ*	Methionine-binding protein	0.156
	–	Sugar ABC transporter ATP-binding protein	0.445
	–	Iron-containing alcohol dehydrogenase family protein	0.408
	*racD*	Amino acid racemase	0.08
	–	CMD domain-containing protein	0.461
	–	Amidohydrolase	0.26
	*cuyA*	D-cysteine desulfhydrase	0.082
	–	Amino acid ABC transporter ATP-binding protein	0.466
	*bisC*	Molybdopterin-dependent oxidoreductase	0.407
	*ssuA*	NitT/TauT family transport system substrate-binding protein	0.421
	*tauA*	ABC transporter substrate-binding protein	0.36
	*narK*	NarK family nitrate/nitrite MFS transporter	0.395
	*ubiX*	UbiX family flavin prenyltransferase	0.185
	*tauC*	ABC transporter permease	0.38
	–	Amino acid ABC transporter permease	0.14
Up-regulated	*glpC*	Anaerobic glycerol-3-phosphate dehydrogenase subunit GlpC	2.6

– represents a putative gene.

*ampH* binds to β-lactam antibiotics (González-Leiza et al., 2011; Henderson et al., 1997). We inferred that *ampH* may play an important role in the bactericidal effect of β-lactam antibiotics. To demonstrate the association between *ampH* and β-lactam antibiotics, we constructed an *ampH* deletion strain (Supplementary Figure S6) and determined the MICs of 12 β-lactam antibiotics for the wild-type and *ampH* knockout strains. Compared with wild-type *P. diazotrophicus*, the *ampH* knockout strain exhibited increased MICs for carbenicillin, piperacillin, cefalotin, cefoxitin, cefuroxime, cefotaxime, ceftazidime, cefepime, aztreonam, and ertapenem by at least 2-, 4-, 8-, 4-, 4-, 4-, 4-, 4-, 4-, and 2-fold, respectively (Table 2). This indicates that *aldB* upregulated *ampH* expression and reduced *P. diazotrophicus* resistance to these antibiotics.

### *acoR* is associated with antibiotic susceptibility

Since the *acoR* gene is located directly upstream of the *aldB* gene in the genome, we hypothesize that these two genes are associated. To demonstrate the association between *acoR* and β-lactam antibiotics, we constructed an *acoR* deletion strain (Supplementary Figure S1) and determined the MICs of 12 β-lactam antibiotics for the wild-type and *acoR* knockout strains. Compared with wild-type *P. diazotrophicus*, the *acoR* knockout strain exhibited increased MICs for carbenicillin, piperacillin, cefalotin, cefoxitin, cefuroxime, cefotaxime, ceftazidime, cefepime, aztreonam, and ertapenem by at least 2-, 4-, 4-, 8-, 4-, 8-, 8-, 4-, 4-, and 2-fold, respectively (Table 2). This indicates that *acoR* is associated with some β-lactam antibiotics and reduces *P. diazotrophicus* resistance to these antibiotics.

### *acoR* reduces antibiotic resistance by upregulating *aldB*

**Figure 3:**
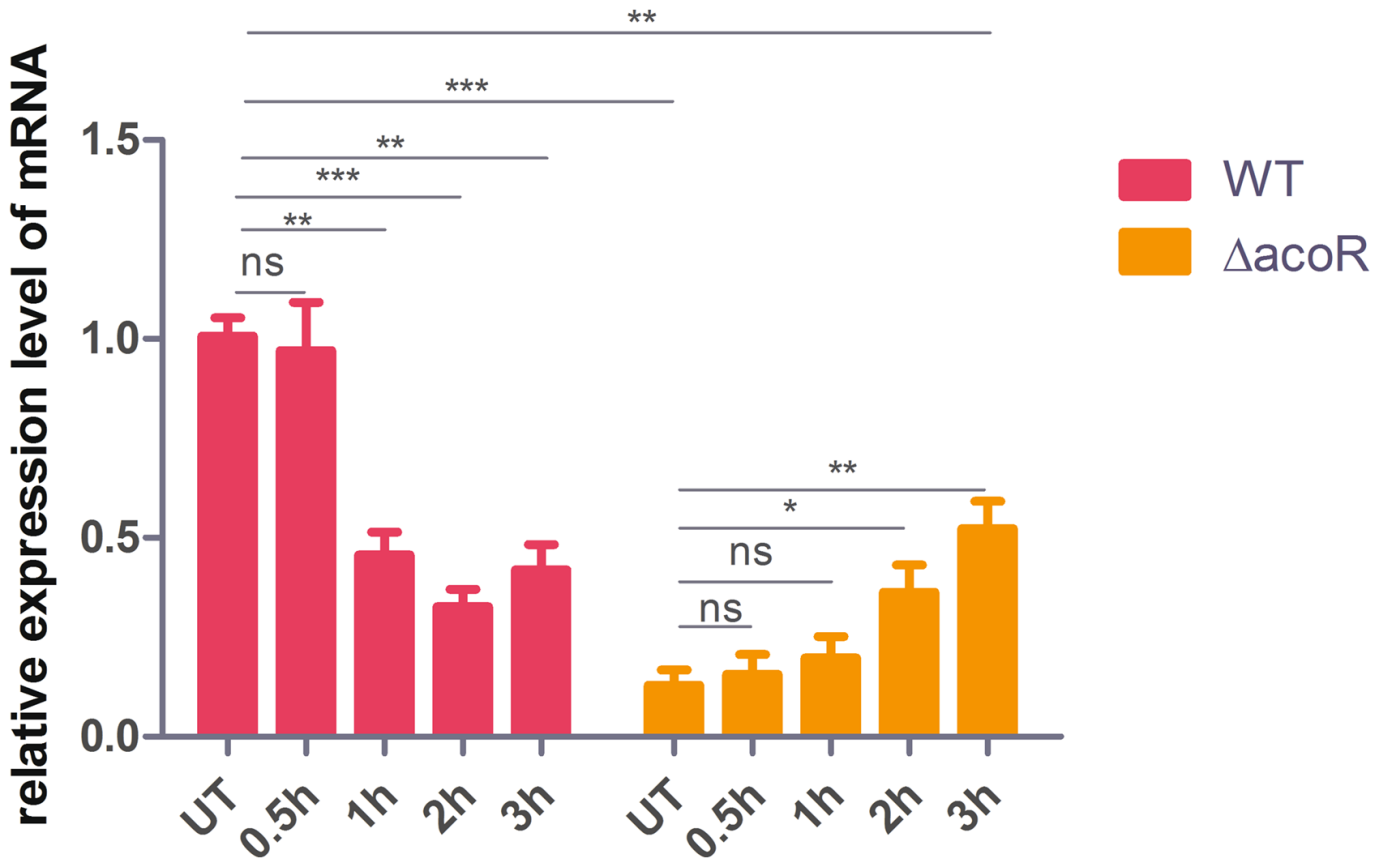
Comparison of relative *aldB* expression levels in wild-type and *acoR* knockout strains of *P. diazotrophicus* following simultaneous stimulation with 2 × MIC ceftazidime for 0.5 h, 1 h, 2 h, and 3 h. WT represents the wild-type strain, △*acoR* represents the *acoR* knockout strain, and UT indicates no treatment. Data represent the mean ± standard deviation of five sample results. * indicates *p* < 0.05; ** indicates *p* < 0.01; *** indicates *p* < 0.001; ns indicates no statistically significant difference.

To demonstrate that *acoR* regulates *aldB* expression, qPCR was used to measure *aldB* mRNA levels in the wild-type and *acoR* knockout strains. The *E* value of *aldB* is 101.5%, which falls within the ideal range (90–110%) for qPCR primer efficiency (Supplementary Figure S7). The melting curve of *aldB* shows a single peak, indicating that the primer for *aldB* has good specificity (Supplementary Figure S4). Compared with wild-type *P. diazotrophicus*, *aldB* mRNA levels in the *acoR* knockout strain were significantly reduced (Figure 3). This indicates that *acoR* upregulated *aldB* expression and reduced *P. diazotrophicus* resistance to these antibiotics.

### The sequence where AcoR binds to the *aldB* promoter

**Figure 4:**
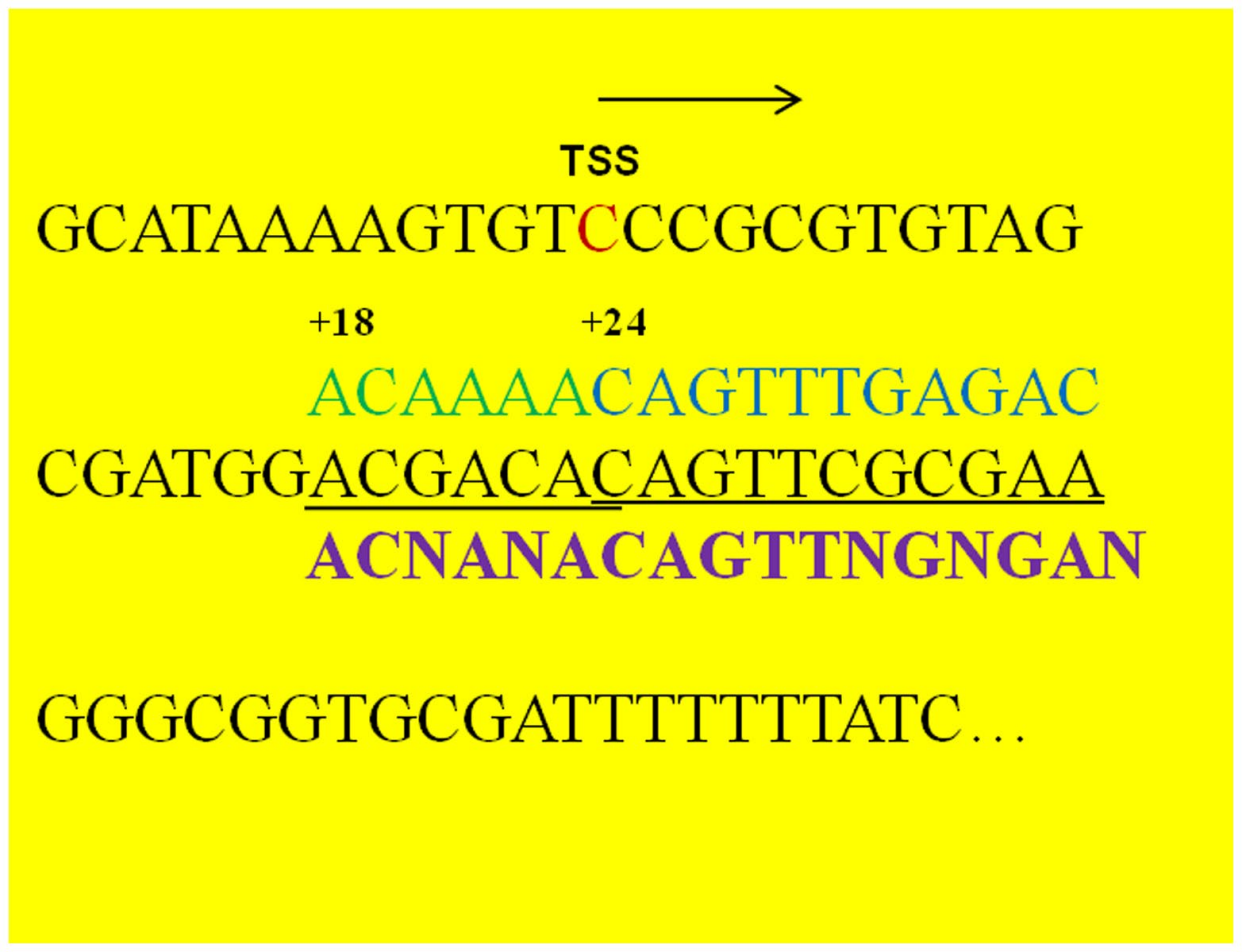
Nucleotide sequence downstream of the *aldB* transcription start site. TSS refers to the transcription start site identified by transcriptomic analysis. Arrows indicate the direction of transcription. The nucleotide sequences in black font (including the TSS) represent the upstream sequence of the translation initiation codon of *P. diazotrophicus aldB*, the nucleotide sequences in green and blue fonts represent the reported binding sequences of the AcoR protein from other bacterial species, the underlined part indicates the potential binding region of AcoR protein in *P. diazotrophicus*, and the sequences in purple font represent the binding motifs of the AcoR protein.

The upstream activating sequences (UAS) sequence (CAGTTTGAGAC) of the *aco* operon is the most likely recognition and binding site for the AcoR protein (Ali et al., 2001). In this study, we aligned this UAS with the sequence upstream of the *aldB* translation start site (ATG) and observed that a similar sequence (CAGTTCGCGAA) was present at position +24 downstream of the *aldB* transcription start site (Figure 4). Additionally, we aligned the sequence upstream of ATG with the AcoR-binding site (GACAAAACGAGACAGATGTCTCATTTTGTC) reported by Peng et al. (2020) and identified a similar sequence (ACGACAC) (Figure 4). The terminal C of this sequence overlaps with the initial C of CAGTTCGCGAA, indicating that ACGACACAGTTCGCGAA may be a recognition site for AcoR in *P. diazotrophicus*. The potential AcoR-binding motifs is ACNANACAGTTNGNGAN.

### AcoR directly binds to the *aldB* promoter

**Figure 5:**
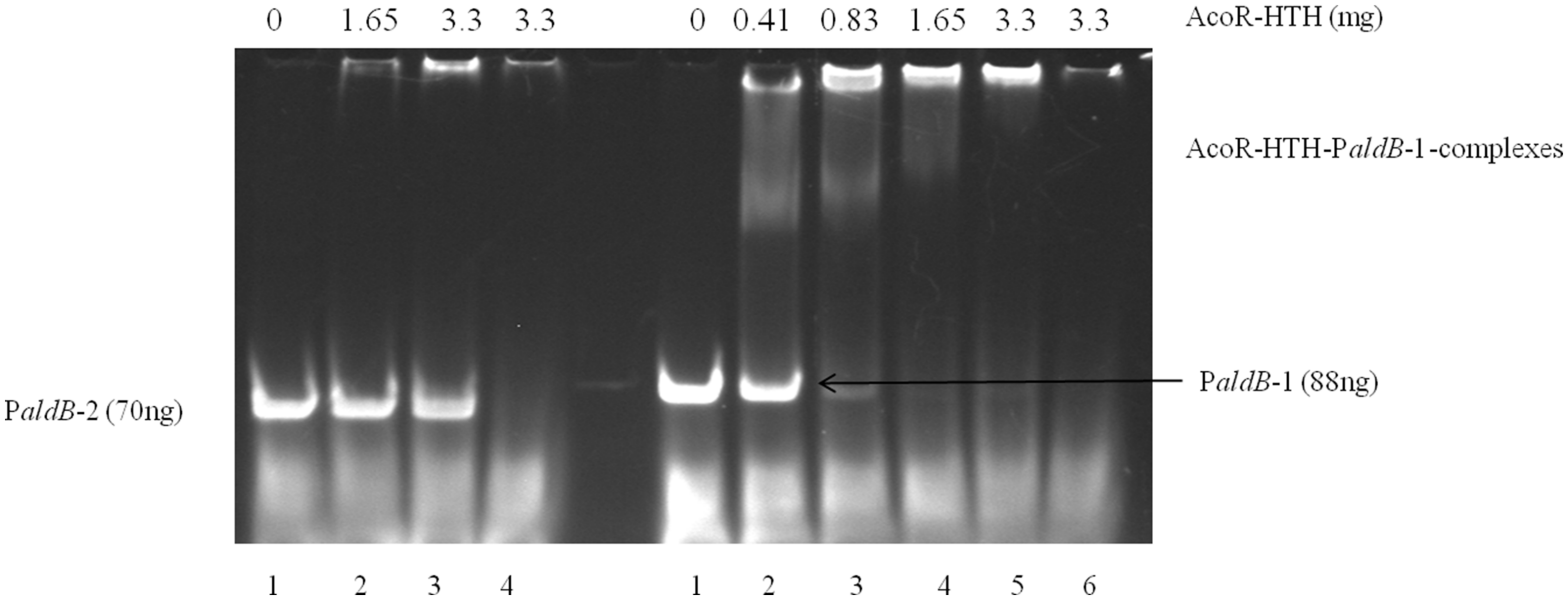
EMSA of AcoR-HTH protein with the *aldB* promoter region. In the left panel of the figure, only probe P*aldB*-2 was added to Lane 1 with no AcoR protein included. Different concentrations of AcoR protein were added to probe P*aldB*-2 in Lanes 2–3. The highest amount of AcoR protein was added to Lane 4, while no probe P*aldB*-2 was included. In the right panel of the figure, only probe P*aldB*-1 was added to Lane 1 with no AcoR protein included. Different concentrations of AcoR protein were added to probe P*aldB*-1 in Lanes 2–5. The highest amount of AcoR protein was added to Lane 6, while no probe P*aldB-1* was included.

To further confirm that AcoR directly binds to the *aldB* promoter and regulates *aldB* expression, we performed EMSA experiments. As shown in the right panel of Figure 5, for the P*aldB*-1 promoter containing the predicted binding site, distinct shifted bands were clearly observed in lanes 2–5. In contrast, no shifted bands were detected in lanes 2 and 3 of the left panel of Figure 5, which corresponds to the P*aldB*-2 promoter lacking the predicted binding site. These results indicate that the predicted binding site is highly likely to be the recognition and binding site of AcoR, which strongly supports the conclusion that AcoR directly binds to the *aldB* promoter to regulate *aldB* expression.

### *aldB* enhances ceftazidime resistance

**Figure 6:**
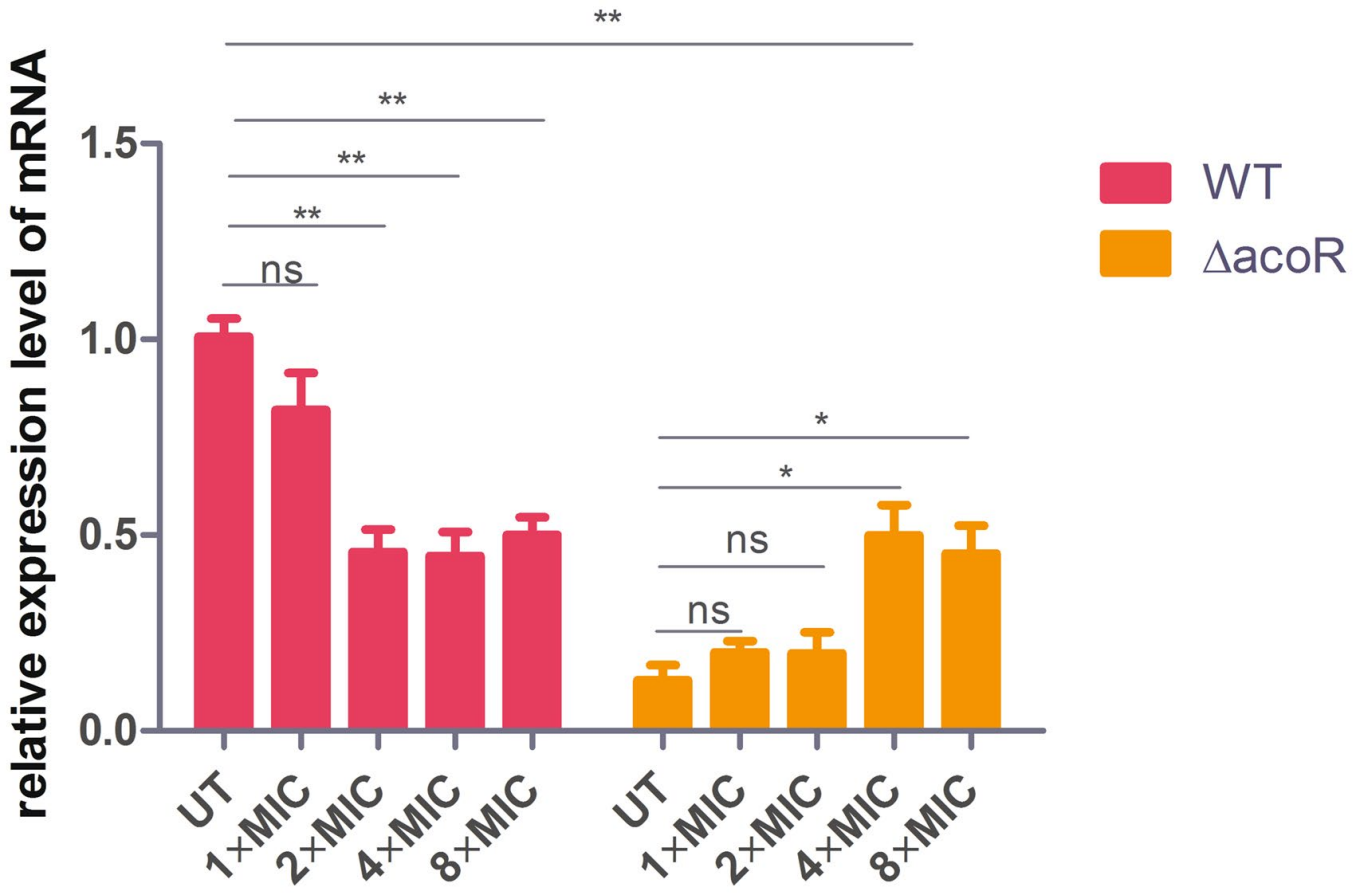
Comparison of relative *aldB* expression levels in wild-type and *acoR* knockout strains of *P. diazotrophicus* following simultaneous stimulation with ceftazidime at different MIC concentrations for 1 h. WT represents the wild-type strain, △*acoR* represents the *acoR* knockout strain, and UT indicates no treatment. Data represent the mean ± standard deviation of five sample results. * indicates *p* < 0.05; ** indicates *p* < 0.01; ns indicates no statistically significant difference.

To assess the role of *acoR*-regulated *aldB* pathway in *P. diazotrophicus* in response to ceftazidime stress, we treated both the wild-type and *acoR* knockout strains with ceftazidime for the dose-dependent and the time kinetic experiment. qPCR was performed to measure *aldB* mRNA levels in the untreated wild-type, untreated *acoR* knockout, ceftazidime-stimulated wild-type, and ceftazidime-stimulated *acoR* knockout strains. First, to assess the alterations in *aldB* under ceftazidime stimulation, we compared *aldB* expression levels between the untreated and ceftazidime-stimulated wild-type strains. Compared with the untreated wild-type strain, the expression level of *aldB* in the ceftazidime-stimulated wild-type strain was significantly reduced under the following two sets of conditions: first, at 2 × MIC with incubation times of 1 h, 2 h, and 3 h; second, at an incubation time of 1 h with concentrations of 4 × MIC, and 8 × MIC (Figures 3, 6). The result showed that under ceftazidime stimulation, the expression of *aldB* decreased, which suggests that *aldB* is involved in the tolerance to ceftazidime.

To further evaluate whether *acoR* is involved in ceftazidime-induced *aldB* downregulation, we compared *aldB* expression levels between the untreated *acoR* knockout and ceftazidime-stimulated *acoR* knockout strains. Compared with the untreated *acoR* knockout strain, the ceftazidime-stimulated *acoR* knockout strain did not significantly upregulate the expression of *aldB* at 2 × MIC for 0.5 h and 1 h, but showed significant upregulation at 2 h and 3 h (Figure 3). Compared with the untreated *acoR* knockout strain, the ceftazidime-stimulated *acoR* knockout strain did not significantly upregulate the expression of *aldB* at 1 × MIC for 1 h, but exhibited significant upregulation at 4 × MIC and 8 × MIC (Figure 6). The result indicates that *acoR* may not be involved in ceftazidime-induced *aldB* downregulation.

## Discussion

AmpH exhibits weak β-lactamase activity against nitrocefin (González-Leiza et al., 2011). If AmpH is a β-lactamase, *aldB*-mediated *ampH* expression may enhance β-lactam antibiotic resistance, in contrast to *aldB*-induced reduction in resistance in *P. diazotrophicus*. Therefore, we concluded that *ampH* does not function as a β-lactamase in *P. diazotrophicus*. *ampH* may serve as an auxiliary drug target for certain β-lactam antibiotics. The *aldB* knockout reduced *ampH* expression, thereby reducing the number of binding sites for β-lactam antibiotics. This reduces the bactericidal effect and increases the resistance of the *aldB* knockout strain to these antibiotics. However, the mechanism by which *ampH* reduces resistance to β-lactam antibiotics requires further exploration.

This study indicates that *aldB* in *P. diazotrophicus* is associated with some β-lactam antibiotics, inconsistent with the report by Wang et al. (2024), who observed no such association. We observed that the complete genomes of *L. reuteri* TD1 and JCM 1112 lack *ampH*, indicating that its absence may have caused this discrepancy.

AcoR upregulates *aldB*, unlike Fis that downregulates *aldB* (Xu and Johnson, 1995), indicating that the members of the Fis family of transcriptional regulators can regulate *aldB* through different mechanisms. Central-domain activators interact with σ^54^ holoenzyme through DNA looping after binding to the enhancer, known as UAS or downstream activating sequences (DAS). Unlike other σ^54^-dependent genes, *rocG* lacks the UAS; instead, its expression depends on DAS that is located downstream of the *rocG* transcription start site (Belitsky and Sonenshein, 1999). Similarly, AcoR enhanced *aldB* expression by binding to DAS.

In the majority of cases, mutation-mediated resistance to ceftazidime is because of the overproduction of chromosomally encoded inducible AmpC β-lactamase (Livermore, 2002). In this study, *ampC* was not detected in *P. diazotrophicus* Pd1, indicating that this bacterium does not resist ceftazidime through induced *ampC* expression but through other pathways. Exposure of bacteria to ceftazidime stress reduced intracellular lipid levels, indicating altered membrane permeability (Peng et al., 2019) a finding supported by our research. In *P. diazotrophicus*, ceftazidime exposure inhibited *aldB* expression, thereby reducing *ampH* expression that affects peptidoglycan synthesis and may alter cell membrane permeability to ceftazidime.

In conclusion, in *P. diazotrophicus*, *aldB* reduced resistance to some β-lactam antibiotics by upregulating *ampH*, whereas AcoR reduced resistance by facilitating *aldB* expression. Under ceftazidime stress, *P. diazotrophicus* downregulated *aldB*, increasing its antibiotic tolerance. The elucidation of the mechanism by which AcoR regulates *aldB* expression provides new clues and directions for the study of bacterial drug resistance.

## Data Availability

The data presented in this study are publicly available. This data can be found here: https://www.ncbi.nlm.nih.gov/sra, accession PRJNA1298522.
