# Supplementary material for: Regulatory relationships among aldB, ampH, and acoR and their impact on β-lactam susceptibility in Phytobacter diazotrophicus
Source: Front Microbiol. 2025 Nov 11;16:1687374. doi: 10.3389/fmicb.2025.1687374 (PMC12648084; doi:10.3389/fmicb.2025.1687374)
Supplement: Supplementary file 1 [file Supplementary_file_1.docx]

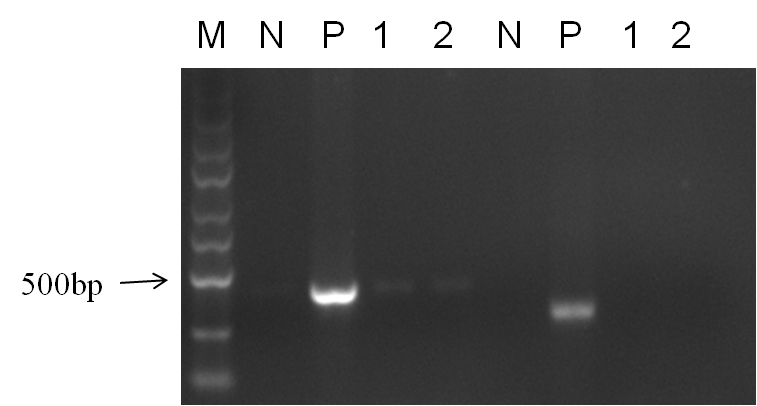


**Figure S1 PCR validation of *aldB* or *acoR* gene knockout in** ***P. diazotrophicus*** M indicates DNA marker，N indicates the negative control, P indicates the positive control, and 1,2 indicate the knockout strains 1,2 respectively. Lanes 2 to 5 indicate *acoR* gene knockout, and lanes 6 to 9 indicate *aldB* gene knockout.


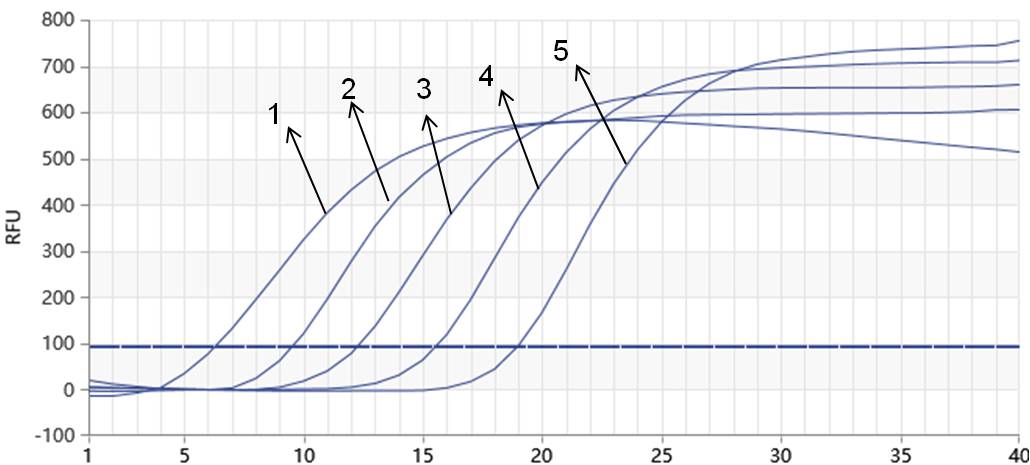


**Figure S2 Amplification curves of the 16S rRNA primer pair for serially diluted cDNA concentrations of the wild-type** ***P. diazotrophicus* Note: amplification curves 1, 2, 3, 4, and 5 represent cDNA concentrations of 1252.4 ng/μL, 125.24 ng/μL, 12.524 ng/μL, 1.2524 ng/μL, and 0.12524 ng/μL respectively**


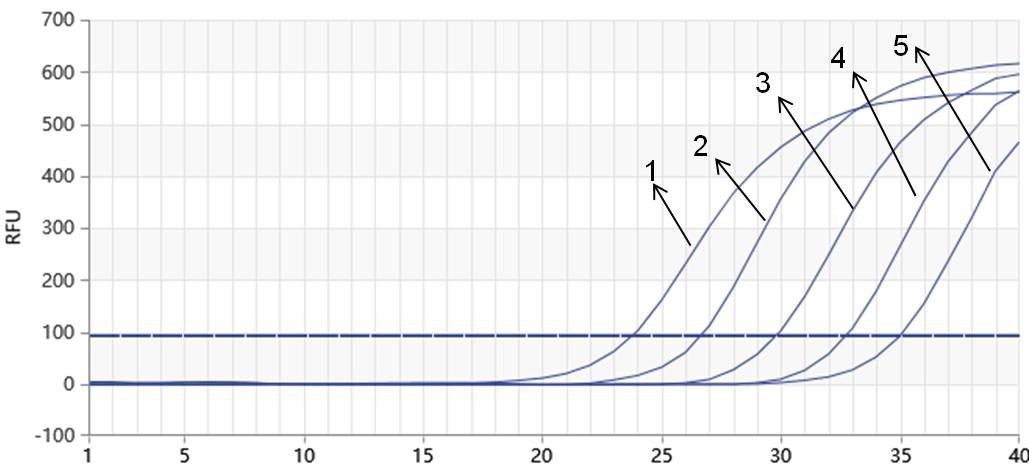


**Figure S3 Amplification curves of the *ampH* primer pair for serially diluted cDNA concentrations of the wild-type *P. diazotrophicus* Note: amplification curves 1, 2, 3, 4, and 5 represent cDNA concentrations of 1252.4 ng/μL, 125.24 ng/μL, 12.524 ng/μL, 1.2524 ng/μL, and 0.12524 ng/μL respectively.**


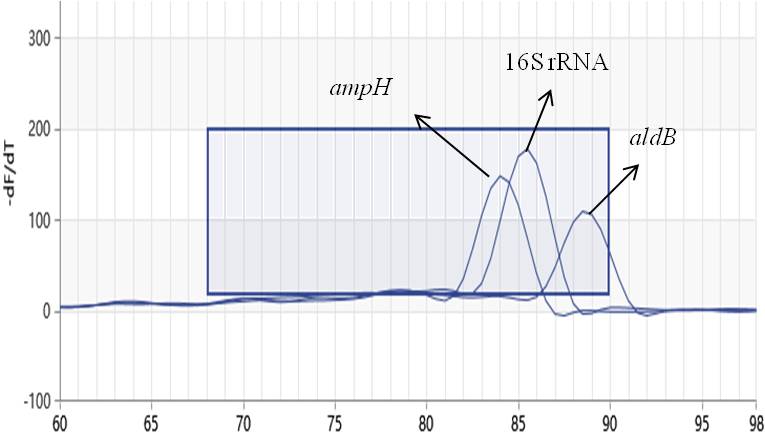


**Figure S4 Melting curve profiles of 16S rRNA, *aldB*, and *ampH***


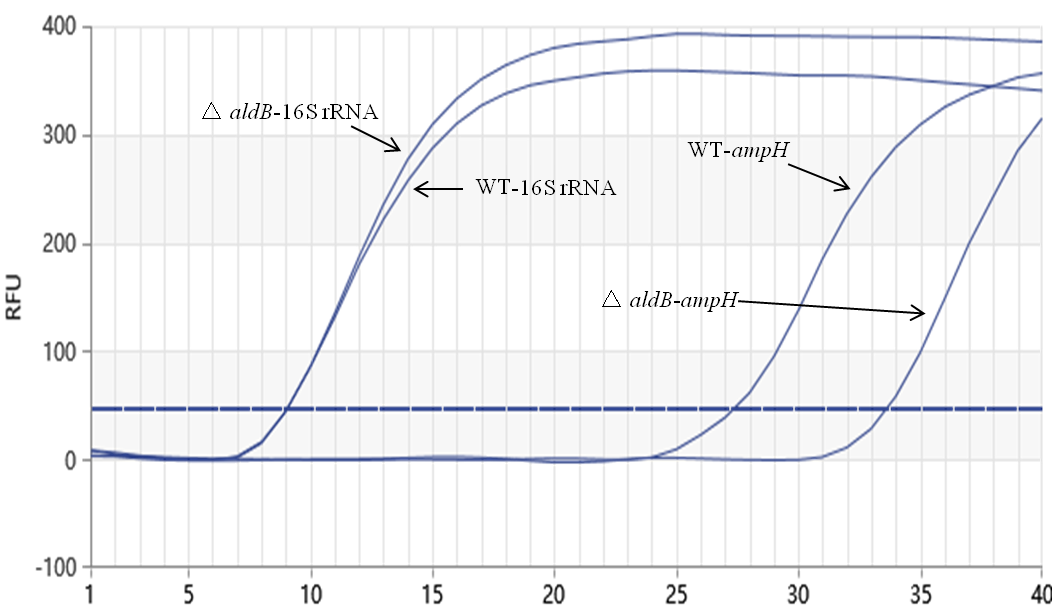


**Figure S5 Real - time amplification curves of 16S rRNA and *ampH* in the wild-type strain, the *aldB* knockout strain of *P. diazotrophicus*,** WT indicates wild strain and Δ*aldB* indicates *aldB* knockout strain


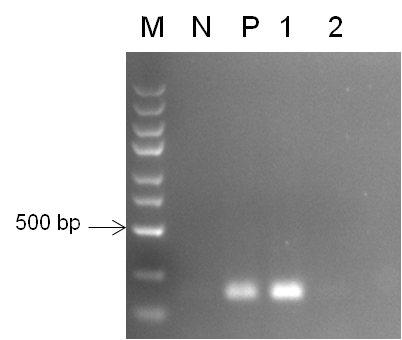


**Figure S6 PCR validation of *ampH* gene knockout in *P. diazotrophicus*** M indicates DNA marker，N indicates the negative control, P indicates the positive control, and 1,2 indicate WT, *ampH* gene knockout respectively.


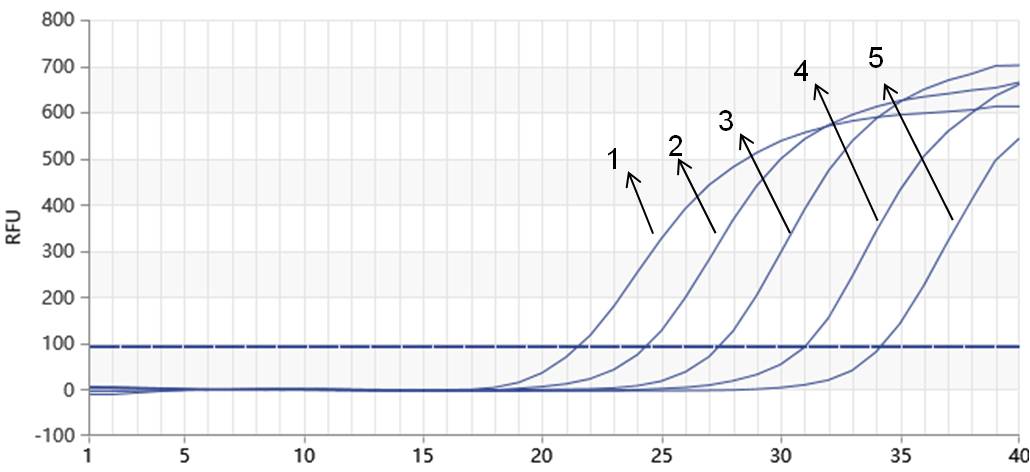


**Figure S7 Amplification curves of the *aldB* primer pair for serially diluted cDNA concentrations of the wild-type *P. diazotrophicus* Note: amplification curves 1, 2, 3, 4, and 5 represent cDNA concentrations of 1252.4 ng/μL, 125.24 ng/μL, 12.524 ng/μL, 1.2524 ng/μL, and 0.12524 ng/μL respectively.**
